# Walking Football During Ramadan Fasting for Cardiometabolic and Psychological Health Benefits to the Physically Challenged and Aged Populations

**DOI:** 10.3389/fnut.2021.779863

**Published:** 2022-01-11

**Authors:** Sueziani Binte Zainudin, Dee Dee A. Salle, Abdul Rashid Aziz

**Affiliations:** ^1^Endocrinology Service, Department of General Medicine, Sengkang General Hospital, Singapore, Singapore; ^2^School of Medical and Health Sciences, Edith Cowan University, Perth, WA, Australia; ^3^Sport Science and Sport Medicine, Singapore Sport Institute, Sport Singapore, Singapore, Singapore

**Keywords:** intermittent fasting, walking football, well-being, social activity, health benefits

## Abstract

Concurrent exercise and intermittent fasting regimens for long periods have been shown to enhance cardiometabolic health in healthy individuals. As exercise and fasting confer health benefits independently, we propose that Muslims who are fasting, especially those experiencing health and clinical challenges, continually engage in physical activity during the Ramadan month. In this opinion piece, we recommend walking football (WF) as the exercise of choice among Muslims who are fasting. WF can be played by any individual regardless of the level of fitness, skills, and age. WF has been shown to elicit cardiovascular and metabolic stress responses, which are suitable for populations with low fitness levels. Most importantly, WF has the inherent characteristics of being a fun team activity requiring social interactions among participants and, hence, likely to encourage long-term consistent and sustainable participation.

## Introduction

The search for a dietary intervention with the most optimal health benefits is ongoing. One such intervention that is gaining popularity is intermittent fasting. Intermittent fasting is when energy consumption in the form of food or drinks is interrupted and markedly reduced for a period of time (1). This is commonly practiced in various regimens such as alternate day fasting, 5:2 diet, and time-restricted feeding. Alternate day fasting involves a fasting day alternating with ad libitum “feast day.” On the other hand, the 5:2 diet includes 5 ad libitum feast days followed by 2 days of fasting. Meanwhile, time-restricted feeding uses the concept of limiting the number of hours for eating each day to between 4 and 8 h usually in the daytime while fasting for the remaining hours.

### Fasting and Health

Intermittent fasting alone may lead to cardiovascular and metabolic benefits (2). A meta-analysis found that intermittent fasting reduces body weight by 1.1 to 6.5 kg and improves lipid profile (3). Four- and 6-h time-restricted feedings can result in similar weight loss by 3.2% and reduce insulin resistance by 12–29% in obese individuals (4). Moreover, time-restricted feeding for 5 weeks has been found to reduce systolic and diastolic blood pressure by 11 ± 4 mmHg and 10 ± 4 mmHg, respectively, in men with impaired glucose tolerance (5).

A recent systemic review and meta-analysis also found that Ramadan fasting (RF) improved cardiometabolic risk factors that may confer short-term protection from cardiovascular disease (6). In another systemic review, all fasting regimens revealed strong evidence to support intermittent fasting as a feasible diet to improve glycemia and body composition measures in obese people with type 2 diabetes mellitus (T2DM) within 12–24 weeks (6) and redistribution of abdominal fat, although a follow-up of 12–18 months after intermittent fasting did not show promising results for continued weight loss and improved glycemic control (6). Intermittent fasting has beneficial effects on lipid profile, and it is associated with weight loss and modification of the distribution of abdominal fat in people with obesity and T2DM as well as improvement in the control of glycemic levels (7).

In a systemic review by Horne et al., there were 3 randomized controlled clinical trials of fasting in humans with results published in 5 articles; all of which evaluated the effects of fasting on surrogate outcomes with improvements in weight and other risk-related outcomes. Improvements in weight and other risk-related outcomes were found in the 3 trials. Two observational clinical outcomes studies on humans were found in which fasting was associated with a lower prevalence of CAD or diabetes diagnosis. No randomized controlled trials of fasting for clinical outcomes were identified (8).

In short, chronic intermittent fasting alone can accrue health and metabolic benefits.

## Lifestyle Changes in Ramadan Fasting

During the annual month of Ramadan, Muslims practice consecutive days of intermittent fasting. This religious practice is frequently accompanied by lifestyle changes and includes altered dietary habits and meal timings and differing sleep and rest patterns, with greater emphasis on religious routines such as increased night time prayers (9). In addition, there are regional differences in RF duration due to differences in daylight and night time hours. Given these drastic changes in circadian rhythm, exercising during Ramadan may be even more challenging for Muslims while fasting, especially for those with prior poor physical fitness and pre-existing health issues, and the frail elderly. It is, thus, not surprising that the evidence points toward greatly reduced physical activity and poorer physical fitness level (10) during the RF month.

### Exercise During Fasting

Exercise and diet, either low energy intake or fasting, have positive effects on health through different pathways and mechanisms (11). There is evidence of increased fat metabolism when exercise is performed in the fasting state rather than post feeding, hence supporting the health benefits for exercise performed when fasting (12). To promote and encourage physical activeness during the RF month, we thus propose in this narrative review that low- to moderate-intensity exercise be performed in the form of WF during the daytime near breaking of fast hours in regions with long daytime fasting such as the equatorial region and summer periods. We reasoned that the combination of RF and WF will potentially augment the health benefits of fasting individuals.

Why specifically WF and not some other types of exercise?

## Walking Football (WF)

Walking football is a variant of football first introduced in England in 1932 for players 65 years and older but recently re-emerged in England aimed at providing more exercise and social networking opportunities for older adults, with rapid recognition globally, especially in Europe, Australia, North America, and Asia (13). In WF, players cannot run and are only allowed to walk throughout the match, defined as one foot in contact with the ground at all times (14). There should not be any physical contact between players and tackling is not allowed during WF to avoid injuries; the ball cannot be above head height. It is a skilled activity that could be performed by more physically challenged individuals either due to older age or restricted mobility, or those with medical conditions restricting physical activities, irrespective of gender. WF is usually played on an indoor court or outdoor field space which have dimensions equivalent to a basketball court but smaller than the soccer pitch. The intensity of WF tends to vary widely from low to high, depending on the duration of each match session and age of the participants, with increased intensity for longer matches and older age groups.

### Effects of WF

To our knowledge, there have been only five studies published on the physiological and psychological outcomes measured from WF sessions, as shown in Table 1 (15–19).

**Table 1:** Acute responses and chronic adaptations of studies on walking football.

**Study**	**Subjects' characteristics**	**Duration of session/ match**	**Key variables measured**	**Results of acute responses**	**Results of chronic adaptations**	**Remarks**
Harper et al. (15)	*N* = 17; F 66 ± 6 y	60 min per session of 5- or 7-a- side matches	Mean match peak HR = 95 ± 8% HR_max_ Mean match HR = 76 ± 6% HR_max_ Mean post-match blood lactate = 3.2 ± 1.7 mmol Mean Players' Load = 353 ± 67 au Mean nos. of Change of direction = 95 ± 11 Mean nos. of Accelerations = 13 ± 3 Mean nos. of Decelerations = 30 ± 4 Mean RPE = 13 ± 2 (somewhat hard)	WF elicit a moderate-to-high intensity stimulus; with significant involvement of anaerobic glycolytic contribution (based on blood lactate). Biomechanically, WF exercise session is equivalent to 25 min of normal running football.	Not applicable	Data was collected over 25 sessions. Participants were experienced in playing WF. HR_max_ was estimated from formula (208–0.7 × age).
Ayabe et al. (16)	*N* = 20; M+F 65 ± 5 y 56 ± 9 kg Some with mild metabolic disorders	2 × 5 min of 5-a-side matches	Mean match peak HR = 92 ± 13% HR_max_ Mean match HR = 82 ± 15% HR_max_ Estimated METs = 8.0 ± 1.6 Step rate per min = 85 ± 18 Nos. of plays with ball = 12 ± 4	WF was deemed of vigorous intensity.	Not applicable	HR_max_ was estimated from formula 220—age. METs was estimated from exercise HR rather than measured directly.
Heil et al. (17)	*N* = 22; F 40 ± 10 y 75 ± 17 kg	2 × 15 min per match with 5–10 min break between half	Mean match HR = 77–80% HR_max_ Mean distance covered during match = 1,650–3,500 m Duration where exercise was > 3 METs = 10–20 min	Exercising HR during WF exceed the physical activity intensity threshold for minimizing non-communicable diseases risks.	Not applicable	Environmental conditions: 26–30°C & 85–90% RH. HR_max_ was estimated from formula 220—age.
Reddy et al. (18)	*N* = 11 in WF group and *N* = 9 in CON group; M+F of betw 50–60 y old	1 × 45–60 min session per wk for 12 wk Each session 5-a-side game	Mean match HR = 76 ± 7% HR_max_ Mean distance covered per match = 2,386 ± 309 m Mean RPE = 13 (somewhat hard)	WF was deemed of moderate intensity.	High levels of enjoyment & individuals were keen to participate in WF regularly. Blood pressure showed enhanced improvement in WF vs control group. No differences in cognitive executive functions between groups.	Control group maintained their normal routine.
Arnold et al. (19)	*N* = 10 M 66 ± 7 y 89 ± 9 kg Possessed some comorbidities	1 × 2 h session per wk for 12 wk Each session consisted of several 15–20 min of 5-a-side game	No physiological data was reported	Not applicable	Body fat ↓ 9%. Body fat mass ↓11%. Systolic blood pressure ↑ 4%. Exercise to exhaustion time ↑ 11 % (but with no change in VO_2max_).	No control group was included.

*M, males; F, females; wk, week; METs, metabolic equivalent; WF, walking football; RPE, ratings of perceived exertion; HR, heart rate; HR_max_, maximum heart rate; CON, control; au, arbitrary unit; VO_2max_, maximal oxygen uptake*.

In Table 1, WF matches were mostly played 5-a-side, suggesting the potential association of the number of players with exercise intensity. In this aspect, a smaller number of players could imply greater distance covered and, consequently, a higher level of muscle activation, since individuals are expected to cover a wider ground and be engaged with the play to a greater extent. In addition, a WF match also involves a high volume of turning and twisting and many spurts of immediate change of direction actions (i.e., very short accelerations and decelerations). Indeed, these movements could have positive effects on bone health, which is clearly beneficial to older aged participants (15).

### Cardiovascular Health Benefits of WF

Harper et al. found that participants developed a mean percentage of maximum heart rate of 76 ± 6% during sessions, with a rating of perceived exertion across all sessions at 13 ± 2. Blood lactate significantly increased by ~157% from pre-session to post-session. There were ~100 changes of direction per session. Hence, WF is a moderate- to vigorous-intensity activity (15).

Ayabe et al. found that the average heart rate was 127 ± 20 beats per min (82 ± 14% of the age-predicted maximum HR) in a 10-min game of WF, with a significant association to the number of plays after adjusting for age. The estimated metabolic cost was 8 ± 1.6 metabolic equivalents with a significant correlation to the maximal oxygen uptake, number of plays with a ball, and stepping rate. Hence, cardiorespiratory responses could be above the desirable levels of exercise prescribed in middle to old-aged adults with mild metabolic disorders (16). However, the authors found that their study was limited by the lack of familiarity of participants to WF, which could account for physiological stress and change with experience.

Heil et al. found that mean relative HRs exceeded the 65% threshold for improving cardiovascular fitness for both teams competing in a match. Both teams also maintained an average metabolic intensity that was statistically similar to the 3.0 MET threshold that decreases one risk for non-communicable diseases and walked an average of 2.2–2.4 km/match. Hence, this is supportive evidence for competitive WF being of sufficient intensity to promote positive changes in both cardiovascular and metabolic fitness in Southeast Asian women (17).

Arnold et al. studied a population with medical conditions such as hypertension, T2DM, knee osteoarthritis, spinal stenosis, atrial fibrillation, bronchitis, and other medical comorbidities with results that prove to be beneficial, including a significant reduction of 11% in body fat mass and in percentage body fat of 9%, and improvement in other anthropometric measures such as 2% reduction in whole body mass, 4% increase in lean body mass, and 3% reduction in body mass index (BMI). However, there was a significant increase in systolic blood pressure by 4% after the 12-week WF program, attributed to lack of medication adherence (19).

In contrast, Reddy et al. found significant greater improvement in blood pressure for players when compared to the control group (18).

In summary, playing WF has been shown to elicit sufficient exercise intensity and duration that would promote cardiovascular fitness, muscular health, and possibly bone health among regular participants of the game.

There are only two studies examining the training-induced adaptations as a result of chronic WF exercise (Table 1). The study of Arnold et al. showed that 12 weeks of WF (2 h per session once a week) among the elderly has had a positive impact on lowering body fat percentage and increasing exercise tolerance (19). However, the study was limited because no physiological data during the WF sessions were reported, and no control group was included in the study (18). The study of Reddy et al. on playing WF once a week between 45 and 60 min, on the other hand, did not show any clinical or health improvements in WF participants relative to controls, although a small positive impact on blood pressure was noted in the WF group (16). Nonetheless, the limited prevailing data on acute responses and chronic training-induced adaptations to WF activity seem to support the potential cardiovascular and clinical health benefits of WF when performed consistently or regularly across a prolonged period of time.

### Psychological Benefits of WF

It should be noted that many older adults had reported a dislike for structured exercise programs (20). During WF, the movements are unstructured, unplanned, and varied. Thus, an interesting and important finding in all the five reviewed studies was that all the participants of WF have reported elevated levels of enjoyment and keen participation in this exercise form (20). Indeed, in a recent review on WF, the authors found that almost all participants in this activity believed that WF provides beneficial mental and physical effects (13). The review also reported that the factors valued the most by the participants were the collaborations among team-mates and team identity during game. The participants of the review also found that compared to visiting the gym or engaging in other regimental physical exercise programs, WF is the preferred form of physical activity. It was concluded that WF has a major positive impact on the overall sense of well-being and social connections of participants (15).

Reddy et al. surveyed a group of elderly who played WF for 12 weeks and found a very positive impact from participation with individuals experiencing high levels of excitement and enjoyment when playing (17). They also highlighted that the ability to meet and make new friends contributed to the self-reported overall improvement in their physical health and well-being (17).

In another study, McEwan et al. similarly showed high adherence to participation in a 12-month WF exercise program among middle-aged obese men (21). When evaluated for a personal perspective of the program, social interaction, group interaction to improve health, and new lease of life were the 3 main themes for continued involvement (21). The opportunity to engage in football and the link to a professional football club 21 were the top 2 factors to continued involvement. Those who participated were overweight, sedentary, exhibited blood pressures outside normal ranges, and all but two were hypertensive. Adherence to the program was 90% over 8 weeks, and of those contacted after a year, all had maintained engagement in WF. Hence, WF is a feasible and cost-effective method of recruiting and retaining men aged at least 50 years old to a physical activity program, although attrition is to be expected.

## Discussion

In view of the commonly reported reduced physical activity during Ramadan, we strongly believe that WF would be the ideal choice of physical activity for populations with varying levels of physical fitness, hence, encouraging fasting Muslims to be active and remain physically active thereafter.

No studies have yet been conducted that have specifically examined the chronic effects of concurrent training on WF and RF. However, studies that have examined the impact of chronic exercise training in the fasted state (but non-Ramadan specific fasting) have shown positive outcomes.

Continuous endurance exercise training (3 days per week, progressively from 25 to 40 min at 60–75% HR_max_) for 12 weeks showed a significantly greater reduction in body weight and favorable lipid profile when the endurance training was performed in conjunction with an alternate day fasting regimen relative to performing either alternate day fasting or exercise on their own among obese subjects (22). This study by Bhutani et al. (22) was among the first to show experimental evidence that the combination of alternate day fasting and exercise produces superior changes in clinical and health markers when compared to either modality alone.

Likewise, Edinburgh et al. (23) showed that in obese participants, 6 weeks of chronic exercise (3 sessions per week cycling at 50–55% peak power output) improved insulin sensitivity and increased skeletal muscle glucose transporter type 4 levels by two times more when performed in the fasted state (overnight fasting) relative to being performed in the fed state.

With regard to cardiovascular exercise performance, Stannard and colleagues showed a significantly greater magnitude of improvement of 9.7% in VO_2max_ among healthy participants who underwent 4 weeks of endurance exercise (5 d·wk^−1^, 25–100 min incrementally at 65% VO_2max_) in the overnight fasted state group relative to the 2.5% improvement in VO_2max_ in the fed group (24). Indeed, in their review, Knuiman and colleagues suggested that exercise training in the fasted state can lead to a much greater metabolic stimulus to the working muscles which could amplify training-induced adaptations relative to the same exercise training performed in a well-fed state (25).

While WF does elicit many cardiovascular and metabolic health benefits to participants, we reiterate that the main advantage of WF relative to other forms of exercise and physical activity programs is its inherent social attractiveness. WF is played in small groups of individuals requiring close teamwork to be successful. The integral socially friendly format of this exercise mode fits nicely and naturally appeals to the physically and metabolically challenged older age group. Indeed, it has been argued critically that WF is likely to be a sustainable form of exercise for older adults (18).

### Ramadan Fasting and WF

To our knowledge, there have not been studies on WF during RF. Future studies looking into health outcomes of WF in RF and the non-fasted state as well as studies comparing WF with other low-intensity exercises during RF in an age-adjusted and gender-adjusted manner would be useful to promote exercise in a more physically active Ramadan to reap the maximal health benefits from RF.

## Conclusion

Fasting and exercise can independently provide health benefits. Hence, we hypothesize that the combined effects of RF and WF training will likely show relatively greater cardiovascular and metabolic health benefits than either RF or WF alone. While more studies directly examining the effects of WF on health in the Ramadan fasted state is needed to provide evidence to support our proposed hypothesis, we propose to encourage fasting Muslims to engage in physical activity, specifically in WF, for cardiometabolic and psychological benefits, in keeping with the spirit of discipline and social interactions encouraged in the Ramadan month.

## Data Availability Statement

The original contributions presented in the study are included in the article/supplementary material, further inquiries can be directed to the corresponding author/s.

## Author Contributions

SZ, DS, and AA: contributed to conception and design. SZ: wrote the first draft of the manuscript. SZ and AA: wrote sections of the manuscript. All authors contributed to manuscript revision, read, and approved the submitted version.

## Funding

This work was funded by Sengkang General Hospital for publication fees.

## Conflict of Interest

The authors declare that the research was conducted in the absence of any commercial or financial relationships that could be construed as a potential conflict of interest.

## Publisher's Note

All claims expressed in this article are solely those of the authors and do not necessarily represent those of their affiliated organizations, or those of the publisher, the editors and the reviewers. Any product that may be evaluated in this article, or claim that may be made by its manufacturer, is not guaranteed or endorsed by the publisher.
